# Sound signatures and production mechanisms of three species of pipefishes (Family: Syngnathidae)

**DOI:** 10.7717/peerj.1471

**Published:** 2015-12-03

**Authors:** Adam Chee Ooi Lim, Ving Ching Chong, Chiow San Wong, Sithi Vinayakam Muniandy

**Affiliations:** 1 Institute of Biological Sciences, Faculty of Science, Universiti Malaya, Kuala Lumpur, Wilayah Persekutuan, Malaysia; 2 Department of Physics, Faculty of Science, Universiti Malaya, Kuala Lumpur, Wilayah Persekutuan, Malaysia

**Keywords:** Fish bioacoustics, Feeding click, Cranial kinesis, Stridulation, Wavelet analysis, Scalogram

## Abstract

**Background.** Syngnathid fishes produce three kinds of sounds, named click, growl and purr. These sounds are generated by different mechanisms to give a consistent signal pattern or signature which is believed to play a role in intraspecific and interspecific communication. Commonly known sounds are produced when the fish feeds (click, purr) or is under duress (growl). While there are more acoustic studies on seahorses, pipefishes have not received much attention. Here we document the differences in feeding click signals between three species of pipefishes and relate them to cranial morphology and kinesis, or the sound-producing mechanism.

**Methods.** The feeding clicks of two species of freshwater pipefishes, *Doryichthys martensii* and *Doryichthys deokhathoides* and one species of estuarine pipefish, *Syngnathoides biaculeatus*, were recorded by a hydrophone in acoustic dampened tanks. The acoustic signals were analysed using time-scale distribution (or scalogram) based on wavelet transform. A detailed time-varying analysis of the spectral contents of the localized acoustic signal was obtained by jointly interpreting the oscillogram, scalogram and power spectrum. The heads of both *Doryichthys* species were prepared for microtomographical scans which were analysed using a 3D imaging software. Additionally, the cranial bones of all three species were examined using a clearing and double-staining method for histological studies.

**Results.** The sound characteristics of the feeding click of the pipefish is species-specific, appearing to be dependent on three bones: the supraoccipital, 1st postcranial plate and 2nd postcranial plate. The sounds are generated when the head of the *Dorichthyes* pipefishes flexes backward during the feeding strike, as the supraoccipital slides backwards, striking and pushing the 1st postcranial plate against (and striking) the 2nd postcranial plate. In the *Syngnathoides* pipefish, in the absence of the 1st postcranial plate, the supraoccipital rubs against the 2nd postcranial plate twice as it is pulled backward and released on the return. Cranial morphology and kinesis produce acoustic signals consistent with the bone strikes that produce sharp energy spikes (discrete or merged), or stridulations between bones that produce repeated or multimodal sinusoidal waveforms.

**Discussion.** The variable structure of the sound-producing mechanism explains the unique acoustic signatures of the three species of pipefish. The differences in cranial bone morphology, cranial kinesis and acoustic signatures among pipefishes (and seahorses) could be attributed to independent evolution within the Syngnathidae, which warrants further investigation.

## Introduction

Teleost fishes are known to produce acoustic signals for intraspecific and interspecific communication (Fish & Mowbray, 1970; Kasumyan, 2008). Acoustic signals in fishes are mainly produced by stridulation, swim bladder pulsation, hydrodynamic movement, tendon vibration and air release, a consistent sound pattern (Fine et al., 1997; Kaatz, 2002; Fine & Parmentier, 2015).

Sound productions in seahorses and pipefishes (Syngnathidae) have been described in various behavioural contexts (Fish, 1953; Fish & Mowbray, 1970; Colson et al., 1998; Ripley & Foran, 2007; Anderson et al., 2011; Oliveira et al., 2014; Lim et al., 2015). So far, three kinds of sounds named click, growl and purr have been reported in syngnathids (Colson et al., 1998; Ripley & Foran, 2007; Haris et al., 2014; Oliveira et al., 2014; Lim et al., 2015). In seahorses, the click originates from the cranial supraoccipital and coronet bones (Colson et al., 1998; Oliveira et al., 2014; Lim et al., 2015), whereas the growl and purr are thought to originate from the pectoral girdle based on vibration analysis (Lim et al., 2015).

Studies on the sounds of seahorses have attracted more attention due to the advancement of sound-recording devices as well as signal-processing tools (Haris et al., 2014; Oliveira et al., 2014; Lim et al., 2015). However, there has been very little work done on pipefishes, a close relative of the seahorse, due to their rarity and fragile nature. Pipefishes have been reported to produce click sounds when feeding or under duress (Burkenroad, 1931; Bergert & Wainwright, 1997; Ripley & Foran, 2007). The feeding strikes and sound clicks produced in *Syngnathus fuscus* and *Syngnathus floridae* was described by Ripley & Foran (2007). In the present study, we recorded and analysed the feeding clicks of the estuarine Alligator pipefish, *Syngnathoides biaculeatus*, and two species of Malayan freshwater pipefishes, *Doryichthys martensii* and *Doryichthys deokhathoides* using spectral and time-frequency (or equivalently, time-scale) distributions. The objective of the study was to describe and compare the click signals of the three species and to relate the sound production to cranial kinesis and morphology of the pipefish’s click producing mechanism.

## Materials and Methods

### Experimental setup for sound recording

Six adult *Syngnathoides biaculeatus*, four adult *Doryichthys martensii* and three adult *Doryichthys deokhatoides* with mean heights (±standard deviation) of 19.4 ± 1.0 cm, 12.5 ± 2.0 cm and 10.9 ± 8.5 cm, respectively, were acquired from a local fish hobbyist shop and kept in separate aquariums (by species) for four weeks prior to the experiment. Experiments with *Syngnathoides biaculeatus* were conducted in an acoustic dampened tank (160.0 cm × 100.0 cm × 45.0 cm) filled with seawater, while experiments with either *Doryichthys martensii* or *Doryichthys deokhathoides* were conducted in smaller acoustic dampened tanks (60.0 cm × 45.0 cm × 40.0 cm) filled with freshwater. Both marine and freshwater tanks were lined on the inside with 1-inch polystyrene foam and air-filled packing wraps, with the tank bottom filled with sand to reduce resonance and reflection (Wysocki & Ladich, 2002). Each experimental tank was placed on a 2-inch thick foam block to further reduce resonance from background noise.

Sound recordings of individual pipefishes were conducted one at a time, over a period of two weeks. The alligator pipefish was first confined in a plastic mesh cage (30.0 cm × 20.0 cm × 45.0 cm; 0.3 cm mesh opening) placed inside the seawater acoustic tank and allowed to acclimatize for 48 h before sound recordings were made. To induce feeding clicks, the pipefish inside the mesh cage was fed with live poecilid fish larvae. Both freshwater pipefishes were not confined in any mesh cage inside the tank, and were fed with live brine shrimp nauplii. All mechanical filters and heaters were shut down two hours prior to sound recordings.

Audio signals emitted during feeding were recorded using a hydrophone (Model C55-F2-LAB: Cetacean Research Technology, Seattle, WA, USA) with a frequency range of 0.006–203 kHz. The hydrophone was omnidirectional with a sensitivity of −165 dBre 1 V/µPa; preamplifier gain: 20 dB connected to a compact flash recorder (Fostex FR-2 24 bit/192 kHz). The hydrophone was placed at mid-water level at the centre of the recording tank. The calculated minimum attenuation distance was 30.1 cm for the saltwater tank and 21.9 cm for the freshwater tank with a minimum resonant frequency of 2353.2 Hz for saltwater and 2802.8 Hz for freshwater. Both distances were below calculated levels based on the equations of Akamatsu et al. (2002). Background noises were pre-recorded as control before the experimental recordings were performed.

All experimental protocols involving the live seahorses were approved by the Institutional Animal Care and Use Committee, University of Malaya (UM IACUC), with Ethics References No. ISB/14/08/2012/ALCO(R). No fish deaths resulted from the experiments, and fish were returned following the experiment to their aquaria housed in the University of Malaya’s Marine Culture Unit.

### Microtomography

Two specimens each of *Doryichthys martensii* and *D. deokhathoides* were studied by microtomography. The pipefish’s head was scanned and analysed using the microtomography equipment (SkyScan 1172, high-resolution micro-CT) and services provided by the Nuclear Agency of Malaysia. The head was mounted onto a polystyrene holder and placed into the scanner to fit into the field of view. Scan layers were set to 10 µm on a 180° rotation to acquire frames. The image frames were collated and reconstructed using a 3D imaging software (CTvox) to determine the cross section and morphological structure of the pipefish’s cranium particularly around the supraoccipital (SOC) region. *Syngnathoides biacuelatus* was not studied due to its large head which could not be mounted onto the scanning equipment.

### Clearing and Staining

The cranial anatomy of alcohol-preserved pipefishes was further examined by using a clearing and double-staining method for bone and cartilage (Dingerkus & Uhler, 1977). For each species, three pipefish heads were cleared in 35.0 ml saturated sodium borate, 65.0 ml distilled water and trypsin powder for 24 h; and then double stained in 100.0 ml 1.0% KOH solution with 1.0 mg Alizarin Red stain for 48 h, and 30.0 mg Alcian Blue, 60.0 ml ethanol and 40.0 acetic acid for 36 h, respectively. Since the whole head could not be viewed in the same field under the microscope, parts of the same head were photographed under a stereo microscope (Leica M125) attached to a digital camera system (Leica MC170 HD; Leica, Wetzlar, Germany). All photos were then stitched together to reproduce the whole pipefish head using the Leica Application Suite (LAS) (version 4.3). Interpretations and descriptions of histological and microtomographic images are primarily based on the terminologies used by Leysen et al. (2011).

### Wavelet analysis

Several studies have used the wavelet transform analysis as a signal processing recognition tool (Selin, Terunen & Tanttu, 2007; Ng, Muniandy & Dayou, 2011; Martinez et al., 2013). The continuous wavelet transform of a signal *X*(s) is defined as (Daubechies, 1992)

\begin{eqnarray*} W(a,t)=\frac{1}{\sqrt{a}}\int \psi \left(\frac{t-s}{a}\right)X(s)d s, \end{eqnarray*}

where the localized function *ψ*(⋅) is called the mother wavelet, *a* is the scale parameter and *t* is the time shift parameter. Scale parameter *a* functions to dilate (*a* > 1) and compress (*a* < 1) the mother wavelet *ψ*, hence creating a time-varying multiscale bases. The scale parameter is inversely proportional to frequency. When the analysed signal is differentiable, a mother wavelet of high regularity will be selected and vice versa. The corresponding ‘energy distribution’ to spectrogram is called wavelet scalogram, and is defined as *S*(*a*, *t*) = |*W*(*a*, *t*)|^2^ (Rioul & Flandrin, 1992). This time-scale representation has an equivalent time-frequency expression that can be obtained by scale-frequency conversion $a=\frac{{f}_{0}}{f}$, where *f*_0_ is the central frequency of the mother wavelet *ψ*. Scalogram has a varying window size at different scales in contrast to fixed-window size of the spectrogram thus preserving the time and frequency resolution.

Prior to analysing sound data, the background signal peak at 30 Hz was removed using wavelet detrending. The raw signal was decomposed into twelve dyadic scale levels using multiresolution decomposition with Daubechies *N* =5 mother wavelet. Then, the 30 Hz signal was reconstructed based on scale level 9 decomposition, and this so-called band-passed interference signal was removed from the raw signal to give the detrended signal.

The wavelet multiresolution was carried out using the Matlab Time-Frequency toolbox developed by Auger et al. (1996). Morlet wavelet of 50 half-length was used to analyse the wavelet scalogram. The scalogram was calculated over 128 analyzed voices and was bounded between 0.001–0.04 Hz normalized frequencies. Squared magnitude of continuous wavelet transform with threshold value over 0.5 was depicted in the scalogram. The energy spectral density that gives the frequency marginal of the scalogram was plotted side by side with the scalogram.

The parameters of the measured sound wave studied included the dominant frequency, which is the highest amplitude value of the measured signal, and the duration of the signal, which is the temporal length of the sound.

### Statistical analysis

The mean of the measured sound parameters was calculated for each species. Sound parameters did not conform to parametric requirements of normality using Shapiro–Wilk’s test and homoscedascity using Levene’s test, even after logarithmic transformation. Hence, differences in the measured sound parameters between three species were tested using Kruskal–Wallis non-parametric test. The Mann–Whitney *U*-test was used to test the differences of the sound parameters of the low frequency component between the congeneric species *D. martensii* and *D. deokhathoides*. All statistical tests were carried out using Statistica 10.0 software (StatSoft Inc., Tulsa, Oklahoma, USA).

## Results

### Sound characteristics

**Figure 1:**
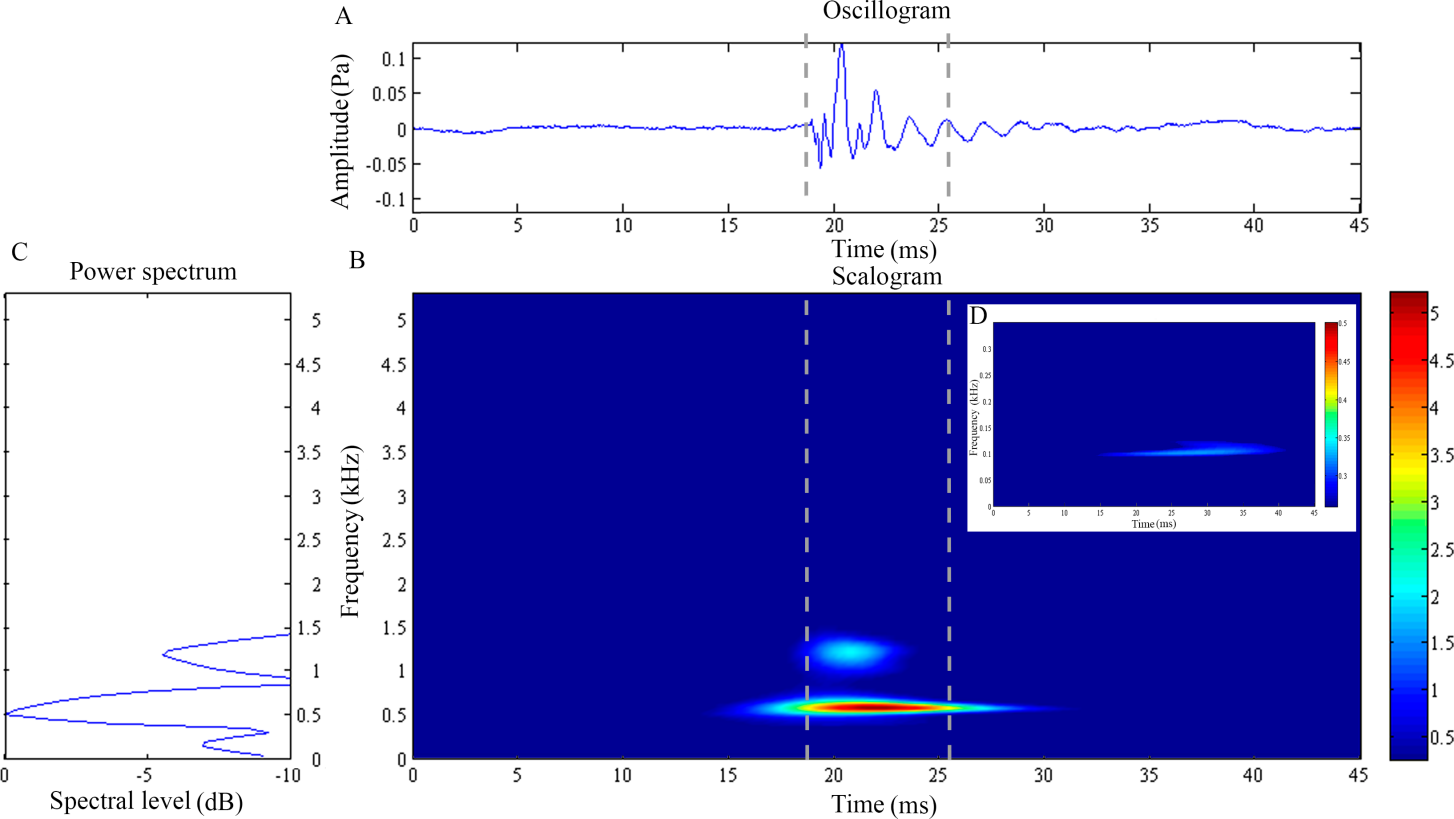
Wavelet analysis results of the feeding click produced by *Doryichthys martensii*. (A) Oscillogram, (B) scalogram (C) energy spectral density and (D) low frequency component of the feeding clicks produced by *Doryichthys martensii*. Vertical lines in (A) and (B) indicate the measured signal duration.

**Figure 2:**
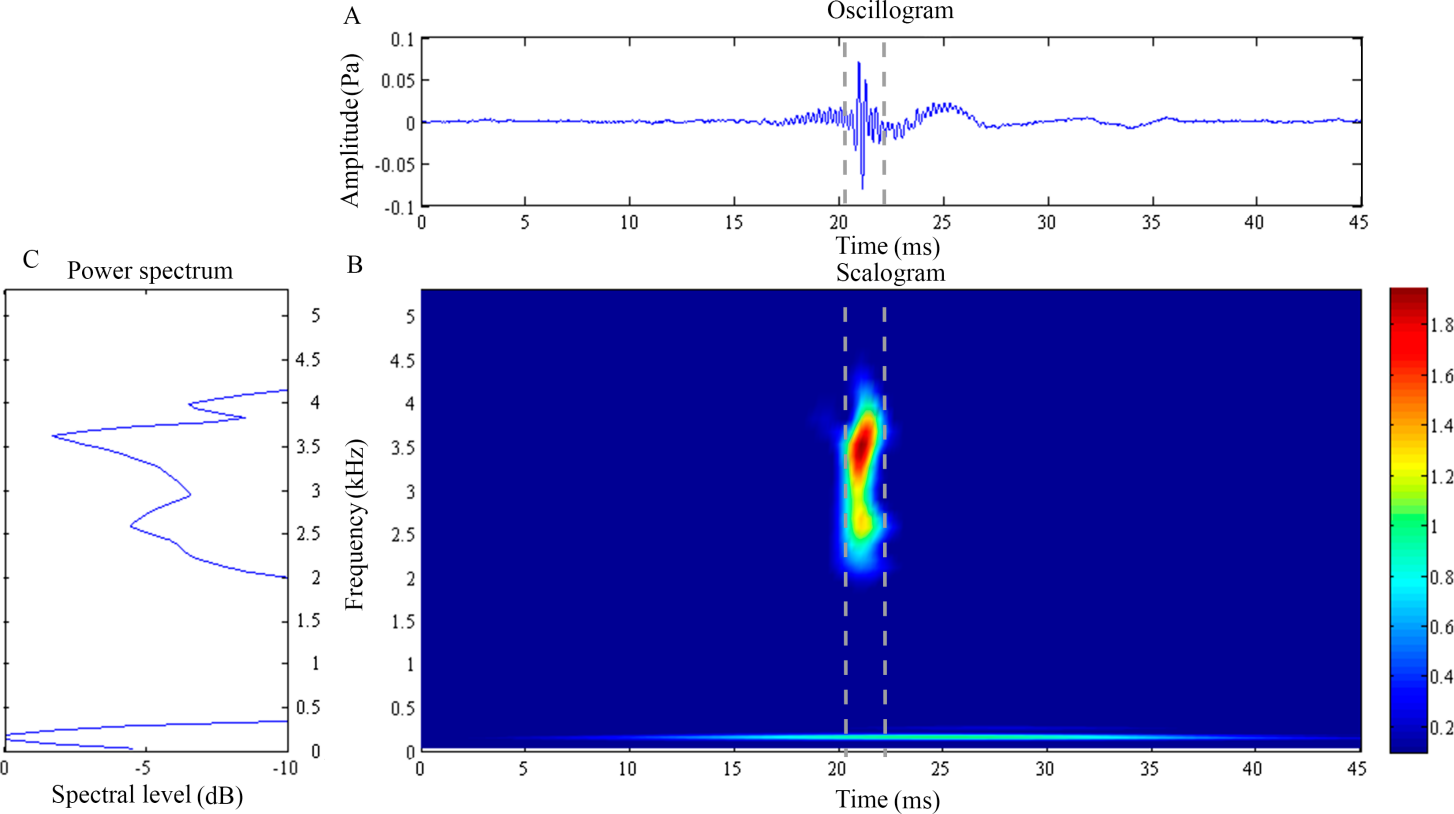
Wavelet analysis results of the feeding click produced by *Doryichthys deokhathoides*. (A) Oscillogram, (B) scalogram and (C) energy spectral density of the feeding clicks produced by *Doryichthys deokhathoides*. Vertical lines in (A) and (B) indicate the measured signal duration.

**Figure 3:**
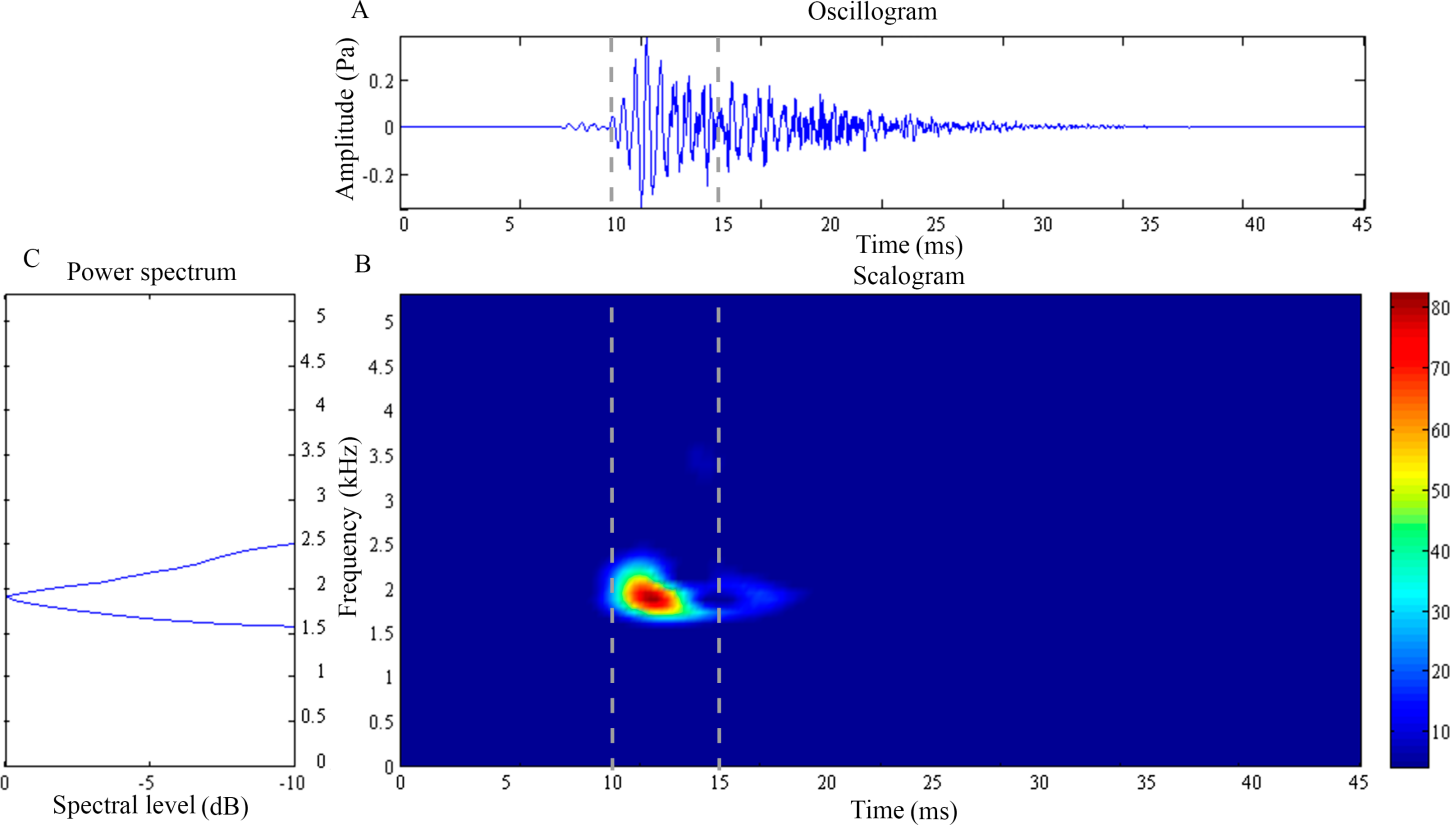
Wavelet analysis results of the feeding click produced by *Syngnathoides biaculeatus*. (A) Oscillogram, (B) scalogram and (C) energy spectral density of the feeding clicks produced by *Syngnathoides biaculeatus*. Vertical lines in (A) and (B) indicate the measured signal duration.

All three species of pipefishes (*Doryichthys martensii*, *Doryichthys deokhathoides* and *Syngnathoides biaculeastus*) produced high frequency, short broadband clicks during feeding strikes (Table 1) (Figs. 1–3). One click per head movement was consistently observed in all three species.

**Table 1:** Feeding click parameters of three species of pipefishes, *Doryichthys martensii*, *Doryichthys deokhathoides* and *Syngnathoides biaculeatus*.

	Characteristics							
	Dominant frequency		Low frequency		High frequency 1		Other components	
Individuals	Mean frequency (kHz)	Mean duration (ms)	Mean frequency (kHz)	Mean duration (ms)	Mean frequency (kHz)	Mean duration (ms)	Mean frequency (kHz)	Mean duration (ms)
*D. martensii*								
DM01	0.58^a^	9.7^a^	0.17^a^	31.7^a^	1.08	4.67	–	–
DM02	0.54	9.2	0.17	28.3	1.12	4.26	–	–
DM03	0.51	11.0	0.17	18.2	1.06	9.25	–	–
DM04	0.51	11.6	–	–	1.03	8.05	–	–
Mean (±SD)	0.54 ± 0.03^A^	10.37 ± 1.10^A^	0.17 ± 0.01^A^	26.05 ± 7.02^A^	1.07 ± 0.04	6.56 ± 2.47	–	–
*D. deokhathoides*								
DD01	2.61^b^	2.35^b^	0.20^b^	16.9^b^	3.68	1.78	1.83	3.6
DD02	2.66	3.46	0.15	9.4	3.85	2.58	2.39	5.3
DD03	2.38	3.09	0.25	4.17	4.00	2.47	1.91	3.1
Mean (±SD)	2.55 ± 0.15^B^	2.97 ± 0.57^B^	0.20 ± 0.03A	10.35 ± 6.40^B^	3.84 ± 0.16	2.28 ± 0.43	2.04 ± 0.30	4.01 ± 1.18
*S. biaculeatus*								
SB01	2.09^c^	3.1^c^	–	–	–	–	–	–
SB02	2.14	2.8	–	–	–	–	–	–
SB03	2.08	3.1	–	–	–	–	–	–
SB04	2.05	4.1	0.82	11.8	–	–	–	–
SB05	2.17	4.5	–	–	–	–	–	–
SB06	2.03	2.7	–	–	–	–	–	–
Mean (±SD)	2.09 ± 0.06^C^	3.40 ± 0.75^B^	0.82 ± 0.01	11.79 ± 0.01	–	–	–	–

**Notes.**

^a,b,c^ All intraspecific differences are not significant.

^A,B,C^ Indicate significant difference among species if different alphabets (alpha = 0.05).

The feeding click waveform of *D. martensii* consists of a single pulsed burst at 0.54 ± 0.03 kHz which decays rapidly (10.37 ± 1.10 ms) (Figs. 1A and 1B). However, its scalogram also revealed a lower energy burst at higher dominant frequency of about 1.07 ± 0.04 kHz but with a shorter duration of 6.56 ± 2.47 ms (Fig. 1B) (Table 1). The power spectrum revealed an additional low frequency component of 0.17 ± 0.01 kHz, with duration of 26.05 ± 7.02 ms (Figs. 1C and 1D). This component was however observed in 77.8% of the total recorded signals (Table 1). The dominant frequency (Kruskal–Wallis test statistic, ${H}_{d f(3),N(18)}^{{\prime}}=4.65$) and duration (${H}_{3,18}^{{\prime}}=2.76$) of all recorded clicks were not significantly different (*p* > 0.05) among four individuals within species. The low frequency component also did not record any significant differences (*p* > 0.05) in its sound characteristics in terms of frequency (${H}_{2,19}^{{\prime}}=1.30$) and duration (${H}_{2,19}^{{\prime}}=4.42$) within species.

The waveform of *D. deokhathoides* revealed symmetrical low-amplitude wave before and after a high-amplitude spike (Fig. 2C). The scalogram revealed a broad band, high-frequency modulation energy burst of between 1.5 and 4.5 kHz which was of short duration (4.01 ± 1.18 ms) (Fig. 2B) (Table 1). This broad band signal appeared to be the merging of two components, 2.04 ± 0.3 and 3.84 ± 0.16 kHz with dominant or modal frequencies of 2.55 ± 0.15 kHz (Fig. 2C). A low frequency component was recorded at 0.20 + 0.03 kHz with an average duration of 10.35 ± 6.40 ms (Figs. 2A and 2B). However, this low frequency component was observed in only 29.2% of the total recorded clicks for the species (Table 1). Both dominant frequency (${H}_{2,14}^{{\prime}}=5.97$) and duration (${H}_{2,14}^{{\prime}}=3.94$) of recorded feeding clicks of *D. deokhathoides* were not significantly different (*p* > 0.05) among the three individuals. Similarly, the low frequency component displayed no significant difference (*p* > 0.05) for frequency (${H}_{2,13}^{{\prime}}=0.70$) and duration (${H}_{2,13}^{{\prime}}=0.36$) within species.

The feeding click of *S. biaculeatus* displays a multimodal sinusoidal waveform of higher amplitude (Fig. 3A) in contrast to the feeding clicks of both *Doryichthys* species. The waveform displays initial small precursor signals, followed by a gradual build-up of large wave signals in two or more overlapping pulses before they followed an extended decay. The double-pulse comprised an initial higher-amplitude (0.2 Pa) pulse of 4 ms, followed very quickly by a second lower-amplitude (0.1 Pa) pulse of 5 ms. The scalogram of *S.biaculeatus* displayed a localised energy burst at a dominant frequency of 2.09 ± 0.06 kHz with an average duration of 3.40 ± 0.75 ms (Fig. 3B), followed by repeated broadband energy releases in the frequency range of 1.8 kHz–6.6 kHz and over a duration of 8.64 ± 0.61 ms (Fig. 3C) (Table 1). Only one recorded click shows an additional low-frequency component which was detected with a dominant frequency of 0.82 kHz and duration of 11.79 ms (not shown in figure). The dominant frequency (${H}_{5,43}^{{\prime}}=2.08$) and duration (${H}_{5,43}^{{\prime}}=2.52$) of all recorded clicks of *S. biaculeatus* was not significantly different (*p* > 0.05) among the six individuals of the same species.

Statistical analysis revealed interspecies differences for both click frequency and duration (Table 1). *Syngnathoides biaculeatus* was significantly different (${H}_{2,94}^{{\prime}}=54.35$) (*p* < 0.05) from *D. martensii* and *D. deokhathoides* in dominant frequency but only *D. martensii* for duration. The dominant click frequency and duration of *D. martensii* and *D. deokhathoides* was significantly different (*p* < 0.05) (Table 1). The recorded frequency of the low-frequency component did not display any significant difference (Mann–Whitney *U*-test: *Z*′ = 1.94; *p* > 0.05) between *D. deokhathoides* and *D. martensii*, except for the duration (*Z*′ = − 2.93; *p* < 0.05) (Table 1).

### Cranial bone morphology and kinesis

**Figure 4:**
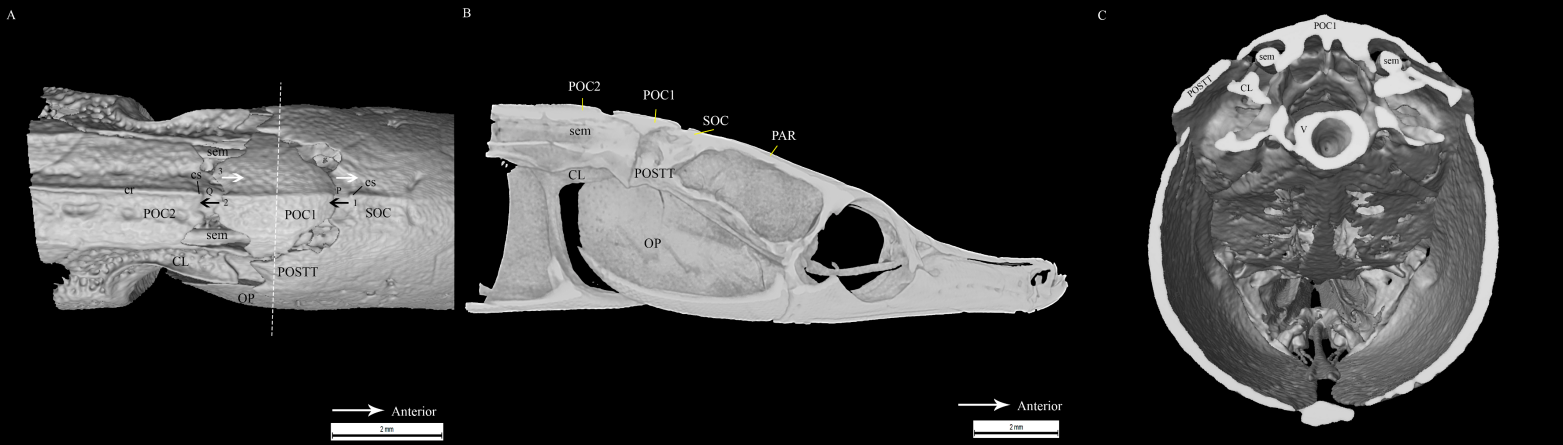
Reconstrcuted tomography image of *Doryichthys martensii* cranium (dorsal, sagittal and transverse aspect) showing morphology of three bones and ancillary structures. Reconstructed tomography images of *Doryichthys martensii* cranium, (A) dorsal aspect, (B) sagittal aspect, and (C) transverse aspect (at POC1, vertical broken line in (A)), showing morphology of bones (CL, cleithrum; OP, operculum; PAR, parietal; SOC, supraoccipital; POC1, 1st postcranial; POC2, 2nd postcranial; POSTT, h post-temporal; V, vertebrae column) and ancillary structures (cr, carina ridge or crest; cs, cranial spine; sem, sesamoid bone in epaxial tendon). The three-bone sound producing mechanism initiated during head flexion is hypothesized here; horizontal arrows indicate (1) sliding movement of SOC beneath POC1, first bone strike at *P*, followed by (2) sliding movement of POC1 above POC2, second bone strike at *Q*, and (3) return of both POC2 and SOC to their original position after head flexion.

All clicks (*n* = 94) were associated with head movement during feeding strikes. Microtomograph images of the head of *D. martensii* features a prominent 1st postcranial plate (POC1) positioned between the supraoccipital bone (SOC) and 2nd postcranial plate (POC2) (Figs. 4A and 4B). The POC1 (equivalent to the coronet in the seahorse) vaguely resembles a blunt arrow head with an anterior end that is slightly grooved and a caudal end that is bifid. The plate rests on respectively the caudal and rostral extensions of the SOC and POC2. Both extensions of the SOC and POC2 are slightly depressed and V-shaped or taper to the tip. All three bones have a dorsal ridge or carina. Only the dorsal carina of the POC1 runs from anterior to posterior end, while the dorsal carina of both SOC and POC2 runs short of reaching their posterior or anterior edge, each ending in a raised, short or blunt spine (Fig. 4A). The lateral section of *D. martenssi* further revealed that POC1 is not fused to, or articulated with the adjacent bones (Fig. 4B). The POC1 shows an arched structure resembling a fused double-chevron in transverse section (Fig. 4C). The long twin sesamoid bones in epaxial tendons (SEM) which run anteriorly from the neck to ligamentously join the SOC are clearly visible beneath the POC1. The arched lateral arms of the POC1 are close to both the post-temporal bone (POSTT) of the cranium and the cleithrum (CL) of the pectoral girdle. The linear system of guided interlocking cranial bone plates which are neither fused nor articulated facilitates bone movements. As the head of the pipefish flexes backwards when the SEM pulls on the SOC, the latter slides beneath the POC1 pushing it towards POC2. The SOC’s carina and raised posterior edge provides the “push” against the POC1 while POC2’s carina provides the “brake.”

**Figure 5:**
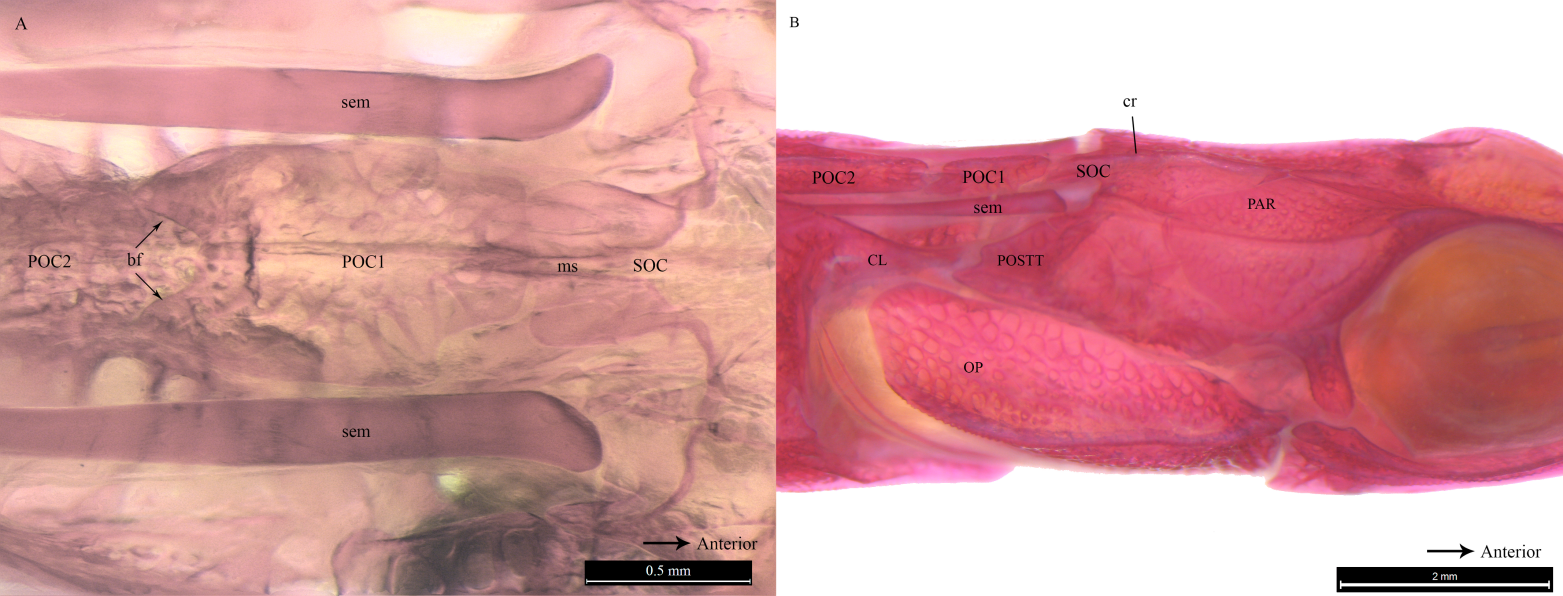
Stained image of *Doryichthys deokhathoides* cranium (dorsal and lateral aspect) showing morphology of three bones and ancillary structures. Alizarin Red stained images of *Doryichthys deokhathoides* cranium, (A) dorsal aspect and (B) lateral aspect, showing morphology of bones (CL, cleithrum; OP, operculum; PAR, parietal; SOC, supraoccipital; POC1, 1st postcranial; POC2, 2nd postcranial; POSTT, post-temporal;) and ancillary structures (cr, carina ridge or crest; cs, cranial spine; sem, sesamoid bone in epaxial tendon; bf, bifid arms of POC1; ms, posterior medial spine of SOC).

Similar to the cranial structure of *D. martensii*, the POC1 of *D. deokhathoides* is visibly present between the SOC and POC2 (Fig. 5A). However, the bone morphology of all three bones differs from that of *D. martensii*. In *D. deokhathoides*, the SOC’s dorsal carina ends caudally as a raised blunt spine, while its inferior caudal extension is expanded like a fan with a long medial spine. Both POC1 and POC2 are narrow bone plates located between the twin SEM which run laterally on both sides. The POC1 has more pronounced caudal bifids than in *D. martensii* (Fig. 5A). The fan-like caudal extension of the SOC of *D. deokhathoides* is tucked underneath the POC1 (Fig. 5B). Similar to *D. martensii*, backward movements of the head would result in the SOC sliding under the POC1, and the latter (bifids) sliding over the POC2.

**Figure 6:**
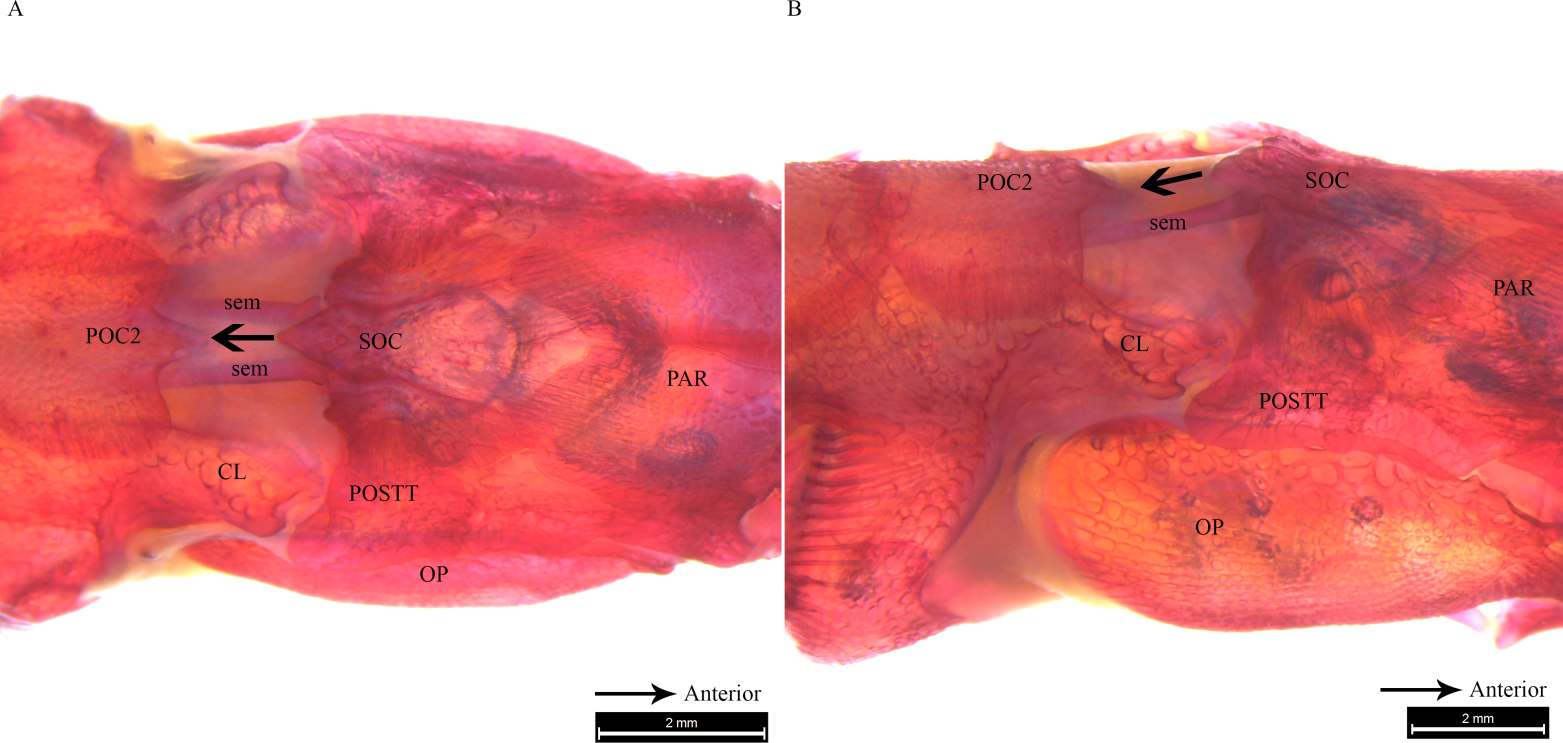
Stained image of *Syngnathoides biaculeatus* cranium (dorsal and lateral aspect) showing morphology of two bones and the sesamoid bones in epaxial muscles. Alizarin Red stained images of *Syngnathoides biaculeatus* cranium (A) dorsal aspect and (B) lateral aspect, showing morphology of bones (CL, cleithrum; OP, operculum; PAR, parietal; POC2, 2nd postcranial; POSTT, post-temporal; SOC, supraoccipital) and the sesamoid bone in epaxial tendon (sem). Note the absence of POC1 as observed in other pipefishes. Arrow indicates the backward movement of SOC which stridulates POC2.

Clearing and staining of the head of *S*. *biaculeatus* revealed the presence of a V-shaped plate at the posterior end of the SOC and on the anterior end of the POC2, just like in *D. martensii*. However, the POC1 plate is distinctly absent leaving a space of about 2 mm between the SOC and POC2 in a fish of 193 cm height (Fig. 6A). Also clearly different from the two *Doryichthys* species is the absence of a dorsal carina and terminal spine on both the SOC and POC2 of *S. biaculeatus*. The twin SEMs are largely exposed between the SOC and POC2. During head flexion, the SOC is pulled backward by the SEM towards POC2. The large, caudal extension (beak-like) of the SOC thus slides over the rostrum of the POC2 which provides the rough stridulating surface (Fig. 6B).

## Discussion

The click signal waveform of *Doryichthys* pipefishes is consistent with that of other species and their close relative, the seahorse, in depicting an initial burst of energy followed by a rapid decay of signal energy (Colson et al., 1998; Ripley & Foran, 2007; Chakraborty et al., 2014; Haris et al., 2014; Oliveira et al., 2014; Lim et al., 2015). However, the signal waveform of *S. biaculeatus* depicts a multimodal sinusoidal waveform unlike the sharp energy spikes recorded in the *Doryichthyes* pipefishes. The dominant frequencies and duration of clicks of all three pipefish species generally displayed no intraspecific differences and are consistent with those reported for *Syngnathus floridae* and *Syngnathus fuscus* (Ripley & Foran, 2007). However, it has been documented that interspecific differences were observed in recorded clicks of other pipefishes such as *Syngnathus floridae* and *Syngnathus fuscus* (Ripley & Foran, 2007). In the present study, all three species are different in their dominant click frequency. However, the click duration is not different between *S. biaculeatus* and *D*. *martensii* but both are different from *D*. *martensii*. Nevertheless, the produced scalograms of all three species displayed species-specific spectral forms which were quantitatively consistent within species. Thus, wavelet analysis together with sound characteristics values could be a useful tool for species differentiation based on sound. The possibility of interspecific differentiation in sound characteristics among seahorse species has also been suggested previously (Lim et al., 2015). Sound diversity within the same family has also been reported in other fish families which may allow species recognition (Amorim, 2006; Kaatz et al., 2010; Fine & Parmentier, 2015). Nevertheless, there are other sources of sound variability such as fish size, ontogeny and sex (Amorim, 2006) which were not covered in the present study. For instance, a negative correlation between frequency peaks and fish size in the seahorse *Hippocampus zosterae* has been reported (Colson et al., 1998), while there are sexual differences in the sound characteristics of *Hippocampus reidi* (Oliveira et al., 2014).

Microtomography and histostaining reveal similarity in the cranial bones associated with sound production in *D. martensii* and *D. deokhathoides* in that a POC1 bone plate is present between the SOC and POC2 plate, depicting a linearly arranged three-bone mechanism (SOC-POC1-POC2). However, this mechanism is modified in *S. biculeatus* where POC1 is clearly absent. Despite the similarity in both *Doryichthys* species, the size and morphology of the associated bones differs between both species which may produce different sound signals or their patterns (see Figs. 2 and 3). In pipefishes, the twin sesamoid bones, epaxial tendons and muscles provide the traction power that pulls the SOC backwards during head flexion (Van Wassenbergh et al., 2008; Leysen et al., 2011). The backward slide of the SOC initiates stridulations and bone strikes between the SOC and postcranial plates behind it. The three-bone mechanism provides two possible successive strikes between the kinetic cranial bones. In the case of *S. biculeatus*, in the absence of the POC1, a stridulatory movement between the SOC and POC2 is produced and there appears to be no bone strike from the sound waveform produced. The much amplified sinusoidal waveform of *S. biaculeatus* feeding click could be attributed to the longer distance between the SOC and the POC2 when the two bones stridulate. Stridulation rather than knocking between cranial bones is more likely given the bone morphology including surface roughness, and the greater distance between bones to generate greater energy.

More evidence of stridulation in *S. biculeatus* is gleaned from the generated waveform (Fig. 3A) which closely resembles that of the blue catfish when the pectoral spine rubs against the pelvic girdle (Mohajer, Ghahramani & Fine, 2015). However, there is a difference in the waveform; it is multimodal in the Alligator pipefish but unimodal in the blue catfish. In the Alligator pipefish, the pulse appears to result from a more forceful backward slide of the SOC over the POC2. The Alligator pipefish’s click waveform in fact agrees with the general description of the stridulatory mechanism which produces an assemblage of irregular transient pulses of a wide range of frequencies (Hawkins, 1993). Waveforms produced by stridulation and forceful strike (knock) between bones and appearing in that order are clearly evident in *D. deokhathoides* (see Fig. 2) consistent with the three-bone sound producing mechanism. Interestingly, the seahorse which makes a single and forceful bone strike also produces an energy burst of high-frequency sound but this involves the highly modified and elevated SOC and POC1 (or coronet) plate (Colson et al., 1998; Leysen et al., 2011; Lim et al., 2015). Thus, the differences in cranial bone morphology explain the different sound signals or their patterns as observed in the pipefishes.

The wavelet analysis also reveals a low-frequency sinusoidal component present in most of the recorded feeding clicks of *D. martensii*, only consistently in one individual of *D. deokhathoides*, and none in *S. biaculeatus*. This low-frequency component has never been reported before. Previous works did not detect this component because the methods employed only revealed the click waveforms (oscillogram) which are superimposed thus masking its presence (e.g., Burkenroad, 1931; Ripley & Foran, 2007). This component is observable in our study where the low frequency component can only be visible in the scalogram and not the oscillogram. The temporal pattern of the low frequency component was found to be different from the dominant higher frequency component. A similar low-frequency component was also reported in the sound click of the tiger-tail seahorse, *Hippocampus comes* by Lim et al. (2015) who suggested that the seahorse’s sound click is actually a compounded sound produced by two mechanisms, one by the cranial bones and the other by stochastic resonance of the pectoral girdle. Interestingly, the temporal pattern of the low-frequency component matches that reported in the tiger-tail seahorse, *Hippocampus comes*, which has a frequency range of 150–200 Hz. Thus, it is also possible that the low-frequency sound component in both *Doryichythes* pipefishes is a secondarily-derived sound produced by the pectoral girdle and stimulated by the cranial bones, given the close proximity of POC1 and cleithrum. In *D. deokhathoides*, these bones are farther apart. However, it is not clear why *S. biaculeatus* does not display this low-frequency signal although one morphological reason could be connected to the absence of the POC1 bone. However, we believe that the more likely reason is that the sliding between the SOC and POC1 bones basically produces a short band signal which may not stimulate resonance of the girdle (Lim et al., 2015).

## Conclusions

In conclusion, the feeding clicks of pipefishes display a localised energy distribution with interspecific differences and unique spectral signatures. The differences in cranial bone morphology (i.e., those associated with sound production) in the three species appear to be an important factor in the production of species-specific signatures. Such varied morphologies and acoustic signatures may benefit species identification. The varied acoustic signatures may have significance in intra- and interspecific communication in pipefishes which however require further work. The differences in both cranial bone morphology and sound signatures among pipefishes (and seahorses) could be attributed to independent evolution within the family. An interesting hypothesis that can be derived from the study is that the three-bone structure (SOC–POC1–POC2) of the sound-producing mechanism in the Syngnathidae is the ancestral feature whereby modifications of it, as observed in both pipefishes and seahorses, have been evolutionary or adaptively derived. Thus, further investigations on the evolution of sound producing mechanisms in the Syngnathidae are warranted.

## Supplemental Information

10.7717/peerj.1471/supp-1 Supplemental Information 1 Sound dataset

## Additional Information and Declarations

### Data Availability

The following information was supplied regarding data availability: Figshare: http://dx.doi.org/10.6084/m9.figshare.1594698.
